# Attachment of *Salmonella* strains to a plant cell wall model is modulated by surface characteristics and not by specific carbohydrate interactions

**DOI:** 10.1186/s12866-016-0832-2

**Published:** 2016-09-15

**Authors:** Michelle Sze-Fan Tan, Sean C. Moore, Rico F. Tabor, Narelle Fegan, Sadequr Rahman, Gary A. Dykes

**Affiliations:** 1 School of Science, Monash University Malaysia, Jalan Lagoon Selatan, 47500 Bandar Sunway, Selangor Darul Ehsan Malaysia; 2 CSIRO Agriculture and Food, 671 Sneydes Road, Werribee, VIC 3030 Australia; 3 School of Chemistry, Monash University, Clayton campus, Wellington Road, Clayton, VIC 3800 Australia; 4 School of Public Health, Curtin University, Perth, WA 6845 Australia

**Keywords:** *Salmonella enterica*, Attachment, Carbohydrate, Pectin, Xyloglucan, Plant cell wall model, Atomic force microscopy, Confocal laser scanning microscopy, Scanning electron microscopy

## Abstract

**Background:**

Processing of fresh produce exposes cut surfaces of plant cell walls that then become vulnerable to human foodborne pathogen attachment and contamination, particularly by *Salmonella enterica*. Plant cell walls are mainly composed of the polysaccharides cellulose, pectin and hemicelluloses (predominantly xyloglucan). Our previous work used bacterial cellulose-based plant cell wall models to study the interaction between *Salmonella* and the various plant cell wall components. We demonstrated that *Salmonella* attachment was favoured in the presence of pectin while xyloglucan had no effect on its attachment. Xyloglucan significantly increased the attachment of *Salmonella* cells to the plant cell wall model only when it was in association with pectin. In this study, we investigate whether the plant cell wall polysaccharides mediate *Salmonella* attachment to the bacterial cellulose-based plant cell wall models through specific carbohydrate interactions or through the effects of carbohydrates on the physical characteristics of the attachment surface.

**Results:**

We found that none of the monosaccharides that make up the plant cell wall polysaccharides specifically inhibit *Salmonella* attachment to the bacterial cellulose-based plant cell wall models. Confocal laser scanning microscopy showed that *Salmonella* cells can penetrate and attach within the tightly arranged bacterial cellulose network. Analysis of images obtained from atomic force microscopy revealed that the bacterial cellulose-pectin-xyloglucan composite with 0.3 % (w/v) xyloglucan, previously shown to have the highest number of *Salmonella* cells attached to it, had significantly thicker cellulose fibrils compared to other composites. Scanning electron microscopy images also showed that the bacterial cellulose and bacterial cellulose-xyloglucan composites were more porous when compared to the other composites containing pectin.

**Conclusions:**

Our study found that the attachment of *Salmonella* cells to cut plant cell walls was not mediated by specific carbohydrate interactions. This suggests that the attachment of *Salmonella* strains to the plant cell wall models were more dependent on the structural characteristics of the attachment surface. Pectin reduces the porosity and space between cellulose fibrils, which then forms a matrix that is able to retain *Salmonella* cells within the bacterial cellulose network. When present with pectin, xyloglucan provides a greater surface for *Salmonella* cells to attach through the thickening of cellulose fibrils.

## Background

Minimally processed fresh produce has been implicated as the major cause of microbial foodborne disease around the world [1]. Bacterial attachment is an important step in the colonization and transmission of pathogens [2, 3]. Processing of fresh produce exposes cut surfaces of plant cell walls (PCW) that are vulnerable to the attachment of human foodborne pathogens, particularly *Salmonella enterica* [4, 5]. Some human pathogens which penetrate the internal tissue after attaching to cut plant surfaces are protected from antimicrobial treatment. Fresh produce harbouring human pathogenic bacteria can subsequently cause foodborne illness when consumed.

PCWs, including those exposed in processed fresh produce, are composed largely of cellulose, pectin and hemicelluloses. Cellulose consists of β (1–4)-linked glucose units that are hydrogen bonded to form a crystalline structure [6]. Xyloglucan, the major type of hemicellulose in the PCW, has a similar backbone to cellulose but is sequentially supplemented with α (1–6)-linked xylose residues as well as occasional galactose and fucose residues [6]. Xyloglucan coats and forms strong cross-links with cellulose microfibrils which prevents microfibril agglutination and also determines the spacing between microfibrils [7]. Pectin, which consists of 90 % α (1–4)-linked D-galacturonic acid residues, forms a matrix around the cellulose-xyloglucan network to provide additional mechanical reinforcement. The pectin network also regulates cell wall porosity and thickness [8]. PCWs play many important roles including their involvement in plant growth, intercellular communication, control of water balance and providing mechanical support to plant cells [9]. In addition, intact PCWs provide a physical barrier against potential pathogens.

The initial step of bacterial attachment to surfaces is generally regarded to be reversible and regulated by weak forces, such as van der Waals forces, electrostatic forces and hydrophobic interactions [10]. This is followed by an irreversible attachment step mediated by strong forces such as covalent bonds, hydrogen bonds and strong hydrophobic interactions [11]. Bacterial attachment can be influenced by many factors, including chemical composition and physical properties of the attachment surfaces [12]. In a previous study [13], we used bacterial cellulose (BC)-based PCW models to investigate the effect of PCW components on the attachment of *Salmonella* strains. PCW models were used instead of native PCW tissues because the heterogeneous composition of the native PCW confounds the study of how individual PCW components affect bacterial attachment. This is because PCW composition differs considerably between species, among plant parts and even for cell types within each cell tissue [14, 15]. Our findings suggested that the presence of specific carbohydrates on PCW components may provide sites for the attachment of *Salmonella enterica* strains [13]. Saggers et al. [16] also suggested that PCW polysaccharides, and particularly pectin, may provide receptor sites for *Salmonella* cells to attach. The role of sugar residues as receptors for the binding of animal pathogenic bacteria to animal cells has long been accepted; for example, it was discovered almost 40 years ago that D-mannose inhibited *Escherichia coli* from binding to human epithelial cells [17, 18]. However, very few studies have focused on the role of sugar residues in plants on the attachment of human pathogens. Our previous study [13] also suggested that varying concentrations of the PCW polysaccharides may have an effect on the structural features of the attachment surface. Physical properties, such as roughness, porosity and hydrophobicity, have also been shown to affect bacterial attachment [19].

Using BC-based PCW models, this study aimed to: i) investigate whether the sugar residues of PCW components (cellulose, pectin and xyloglucan) provide sites for *Salmonella enterica* attachment, and ii) establish the effect of these PCW polysaccharides on the structural properties of the BC-based PCW model and the associated attachment of *Salmonella enterica* to these surfaces. Data obtained from this study suggest that the attachment of *Salmonella* cells to native PCWs were not mediated by carbohydrate interactions but were more likely to be influenced by the physical and morphological characteristics of the attachment surface.

## Methods

### Bacterial strains

*Gluconacetobacter xylinus* ATCC 53524, a cellulose producing strain, and *Salmonella enterica* subspecies *enterica* serovar Enteritidis ATCC 13076 and *Salmonella enterica* subspecies *enterica* serovar Typhimurium ATCC 14028 were obtained from the American Type Culture Collection (ATCC; Manassas, VA, USA). *Salmonella enterica* subspecies *indica* M4 was isolated from lettuce in Malaysia.

*Salmonella* strains were grown aerobically at 37 °C for 18 h on tryptic soy agar (TSA; Merck, Darmstadt, Germany) or in tryptic soy broth (TSB; Merck, Darmstadt, Germany) under shaking incubation (150 rpm) (Lab Companion SK-600 benchtop shaker, Medline, UK). *G. xylinus* ATCC 53524 was cultured statically at 30 °C for 72 h in Hestrin and Schramm (HS) broth (pH 5) containing 2 % (w/v) glucose, 0.5 % (w/v) peptone, 0.5 % (w/v) yeast extract, 0.27 % (w/v) Na_2_HPO_4_ and 0.115 % (w/v) citric acid [20]. HS agar was prepared by adding 1.5 % agar to the HS medium.

### Production of bacterial cellulose-based plant cell wall models

BC-based PCW models were produced using protocols described by Mikkelsen and Gidley [21] with some modifications. A primary inoculum of *G. xylinus* ATCC 53524 was prepared by transferring a colony grown on HS agar into HS broth which was incubated statically at 30 °C for 72 h. The primary inoculum was added to fresh HS medium with or without combinations of pectin and/or xyloglucan to produce a variety of BC composites as shown below:

- Bacterial cellulose (BC) was produced with only HS medium without additional additives.
- BC-Pectin (BCP) was produced by adding 0.5 % w/v apple pectin to the HS medium and 12.5 mM calcium chloride (R&M Chemicals, Malaysia).
- BC-Xyloglucan (BCX) was produced by adding 0.5 % w/v xyloglucan (Megazyme, County Wicklow, Ireland) to the HS medium.
- BC-Pectin-Xyloglucan (BCPX) was produced by adding different combinations of pectin and xyloglucan (% w/v), while an optimal concentration of CaCl_2_ was added to form a low degree of esterification pectin gel according to the amount of pectin added. For example, BCPX composites with 0.1 % and 0.25 % w/v pectin were supplemented with 3 mM and 6 mM CaCl_2_ respectively, regardless of the xyloglucan concentrations.

These BC composites were produced in enclosed plastic containers (1.5 × 1.5 × 1.5 cm^3^) and incubated statically for 72 h. During harvest, BC composites occur as a gelatinous layer on the surface of the HS growth medium. Harvested BC composites (1.5 × 1.5 cm^2^, ~2 mm thickness) were rinsed in 6 mM CaCl_2_ while shaking at 100 rpm for 1 h to remove media components.

### Effect of sugar molecules on the attachment of *Salmonella* strains

Early stationary phase cultures of *S.* Enteritidis ATCC 13076*, S.* Typhimurium ATCC 14028 and *Salmonella enterica* M4 were centrifuged at 5500 *g* (Hettich D-78532, Tuttlingen, Germany) for 10 min at 4 °C. Pelleted cells were then washed twice with phosphate buffer saline (PBS) (pH 7.4) (1st BASE, Singapore) and resuspended in PBS containing monosaccharide sugars (1 % w/v) composing pectin and xyloglucan to a final OD_600nm_ which corresponds to 10^8^ CFU/mL for each isolate. Pelleted cells resuspended in PBS only (10^8^ CFU/mL) served as controls for this experiment. The sugars used (Sigma-Aldrich, USA) were D-Galacturonic acid, D-Mannose, L-Rhamnose, L-Arabinose, D-Galactose, D-Glucose and D-Xylose. Preliminary tests showed that the 1 % w/v concentration used for each monosaccharide was sufficient to examine the effect of the sugars on bacterial attachment and did not affect *Salmonella* viability (results not shown).

Bacterial suspensions prepared with sugar solutions were left at room temperature for 1 h to allow sugar molecules to attach to potential sugar receptors on the *Salmonella* cell surface. Subsequently 4 BC composites [BC, BCP (0.5 %), BCX (0.5 %), BCP (0.25 %) X (0.25 %)] were incubated in the bacterial suspensions for 20 mins. Rinsing of the incubated BC composites with 6 mM CaCl_2_ was carried out for 1 min to remove loosely attached cells. The rinse solution was not plated in this study, however, this is an effective approach which could be used enumerate the bacterial population loosely attached to the BC composites*.* Treated BC composites were then placed in a stomacher bag containing 50 mL PBS and pummeled for 1 min in a stomacher (BagMixer 400; Interscience, France) at a rate of 8 strokes/s. An aliquot of the PBS was serially diluted before spread plating on xylose lysine deoxycholate agar (XLDA; Oxoid, UK) which was used in our previous studies [13, 22, 23] to enumerate the numbers of *Salmonella* cells that were attached to the composites. Attached numbers of bacterial cells were expressed as log CFU/cm^2^ composite. No statistically significant changes in bacterial populations after incubation in the sugar solution were apparent and therefore changes of in numbers of bacteria were highly unlikely to have affected the attachment results.

Preliminary results showed that D-galacturonic acid killed all bacteria at 1 % w/v as the pH of the D-galacturonic acid solution was more acidic (~pH 2.6) as compared to the other sugar solutions which were only slightly acidic (~pH 4.5). For this reason, the pH of the D-galacturonic acid solution was adjusted to pH 4.5 to eliminate any pH effect on bacterial survival during the attachment assays. At 1 % w/v, D-galacturonic acid appeared to have decreased the attachment in the *Salmonella* strains to some of the composites containing pectin and therefore, a higher concentration of D-galacturonic acid at 2 % w/v was used in subsequent attachment assays. Viable counts of *Salmonella* cells were performed by spread plating on XLDA after treatment with 1 % and 2 % w/v galacturonic acid. Another treatment on the *Salmonella* cells was carried out with 1 % w/v sodium metaperiodate, a chemical which cleaves the C-C bond between neighbouring hydroxyl groups in sugars, before incubation with BC composites to further confirm the role of sugar interactions in *Salmonella * attachment to BC composites.

### Microscopy

#### Confocal laser scanning microscopy (CLSM)

CLSM was used to visualize the cross-sectional view of the BC composites in its native hydrated state without any modification. The 3D microstructure of the BC composites [BC, BCP (0.5 %), BCX (0.5 %), BCP (0.1 %) X (0.1 %), BCP (0.1 %) X (0.3 %), BCP (0.1 %) X (0.5 %)] were examined. Different concentrations of the Calcofluor White stain (CW; Sigma-Aldrich, USA) were used to stain the BC composites for 1 min as the presence of pectin and xyloglucan can impede uptake of the stain. BC was stained with 0.01 % w/v CW, BCX and BCPX were stained with 0.02 % w/v CW whereas BCP was stained with 0.1 % w/v CW. After staining, BC composites were rinsed in distilled water for 1 min before adding 10 % w/v potassium hydroxide for another min then viewed at room temperature using a Leica TCS SP5 CLSM (Leica Microsystems, Germany) under a HCX PL APO 100× objective. CW dye was excited by a Diode 405 nm laser and the emitted light was collected from 427 to 477 nm.

In order to ascertain whether *Salmonella* cells can penetrate and attach inside the matrix of the BC composites, *S.* Typhimurium ATCC 14028 was grown for 18 h in Bromothymol Blue (BTB; Sigma-Aldrich, USA) broth which contains 1 % (w/v) casein peptone, 0.5 % (w/v) sodium chloride and 0.0025 % (w/v) BTB dye. *Salmonella* cells grown in and subsequently dyed with BTB broth were pelleted at 5500 *g* for 10 mins at 4 °C and resuspended in PBS. The BCP (0.1 %) X (0.1 %) composite was dyed with CW stain as described above before incubation with BTB dyed *Salmonella* cells for 20 mins. The BTB dye was excited by an Argon 488 nm laser and the emitted light was collected from 707 to 741 nm.

#### Atomic force microscopy (AFM)

BC composites [BC, BCP (0.5 %), BCX (0.5 %), BCP (0.1 %) X (0.1 %), BCP (0.1 %) X (0.3 %), BCP (0.1 %) X (0.5 %)] were air dried for 3 days after harvesting and rinsing. Intermittent contact (AC) mode was used on a JPK Nanowizard 3 AFM (JPK Instruments, Berlin, Germany) with Bruker NCHV model cantilevers (California, USA). AFM height images were analysed using Scanning Probe Image Processor (SPIP) software (NanoScience, USA). The diameter of the microfibrils were determined using the cross section profile tool which extracts height profiles, wherein the width of the peaks represents the width of the microfibrils as illustrated by Cybulska et al. [24]. The mean diameter of microfibrils for each composite was determined from 10 different microfibrils lying on relatively flat surfaces of the BC composites (without large scale features, such as *G. xylinus* cells) chosen from each of 3 images for each type of sample. The average roughness and root mean square (RMS) roughness were determined by choosing 10 relatively flat areas from each of 3 images for every type of BC composite.

### Scanning electron microscopy (SEM)

BC composites [BC, BCP (0.5 %), BCX (0.5 %), BCP (0.1 %) X (0.1 %), BCP (0.1 %) X (0.3 %), BCP (0.1 %) X (0.5 %)] with and without *S.* Typhimurium ATCC 14028 cells attached to them were rinsed in 6 mM CaCl_2_, air dried and fixed with 4 % (v/v) glutaraldehyde (Sigma-Aldrich, USA) in PBS for 40 min. Samples were gradually dehydrated in a series of ethanol concentrations (20 %, 40 %, 60 %, 80 % and 100 % v/v ethanol in water; 10 min for each concentration) before drying the samples in a vacuum desiccator filled with silica gel at room temperature for 3 days. Samples were gold-sputtered using a sputter coater (Q150RS; Quorum, UK) and viewed under a SEM (S-3400 N; Hitachi, Japan).

### Statistical analyses

All experiments were conducted in triplicate with independent cultures. Statistical analysis of results was performed using Statistical Package for the Social Sciences (SPSS) (SPSS Inc., USA) at a 95 % confidence level. A one-way analysis of variance (ANOVA) was performed to determine significance of the effects of different sugar solutions on the attachment of each *Salmonella* strain among the types of BC composites, as well as among the different levels of PCW components used within each type of BC composite. Another one-way ANOVA was carried out to compare the significance of differences in microfibril diameters, as well as surface roughness between different BC composites.

## Results and discussion

### *Effect of sugar molecules on the attachment of* Salmonella *strains*

The BC composites have previously been chemically analysed [13]. When more pectin and/or xyloglucan was present in the HS media, more of these PCW components were incorporated into the BC composites. Of the different types of BC composites, the chemical composition of the bacterial cellulose-pectin-xyloglucan (BCPX) composites (on average with ~33 % cellulose; ~44 % pectin; ~23 % xyloglucan) were most similar to an average native PCW (~25 % cellulose; ~35 % pectin; ~25 % hemicellulose) [24].

Previous experiments [13] suggested that *Salmonella* cells may harbour specific carbohydrate-binding receptors that bind preferentially to complementary carbohydrate molecules. This postulation was made after we observed that the presence of pectin significantly increased the attachment of the *S. enterica* strains to the BCP composite (*p* < 0.05) whereas xyloglucan had no significant effect on *Salmonella* attachment to the BCX composite (*p* > 0.05). Interestingly, among the different BC composites, *S. enterica* strains attached in the highest numbers to the BCP (0.1 %) X (0.3 %) composite which contained 0.3 % w/v xyloglucan in the media regardless of the amount of pectin present. This suggests that xyloglucan at the optimal concentration of 0.3 % (w/v) favours *Salmonella* attachment when in association with pectin in the BCPX composite.

To further investigate the role of sugar molecules on the attachment of *Salmonella* cells to PCW components, the bacterial cells were incubated in sugar solutions prepared from the monosaccharides making up the PCW components. This allows the monosaccharides of interest to potentially block lectins on *Salmonella* cells (which may act as attachment receptors) from associating with complementary sugar molecules (which could act as receptor sites) on the PCW models. If the monosaccharide of interest successfully blocks the attachment receptors on *Salmonella* cells, the attachment of *Salmonella* cells to the BC composites which contain the same monosaccharide would also be inhibited.

An overall comparison indicates that the attachment of *S.* Typhimurium ATCC 14028 (6.90 ± 0.53 log CFU/cm^2^), *S.* Enteritidis ATCC 13076 (6.66 ± 0.45 log CFU/cm^2^) and *S. enterica* M4 (6.57 ± 0.35 log CFU/cm^2^) to the various BC composites were not significantly different from each other (*p* > 0.05). Preliminary experiments showed that the addition of D-galacturonic acid (makes up 90 % of pectin) at 1 % w/v reduced the attachment of all 3 *Salmonella* strains to certain BC composites containing pectin. D-galacturonic acid at 1 % w/v reduced the attachment of *S.* Enteritidis ATCC 13076 to the BCPX composite, *S.* Typhimurium ATCC 14028 to both BCP and BCPX composites and *S. enterica* M4 to the BCP composite respectively, when compared to their attachment without addition of any sugars. This initial finding agreed with the postulation that the *Salmonella* strains may harbour attachment receptors which are specific to D-galacturonic acid residues in pectin. This may also explain the phenomenon of increased *Salmonella* attachment in the presence of pectin as observed in our previous study [13].

**Fig. 1:**
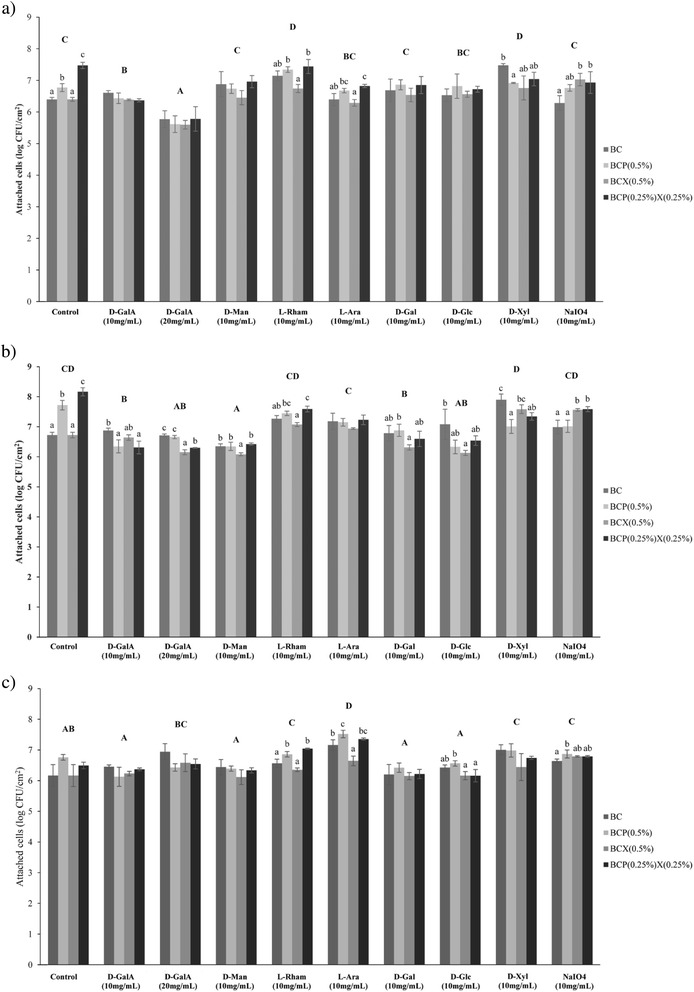
Effect of monosaccharides and sodium metaperiodate on the attachment of *Salmonella* cells to BC composites. **a** *Salmonella* Enteritidis ATCC 13076, **b** *Salmonella* Typhimurium ATCC 14028 and **c** *Salmonella enterica* subsp. *indica* M4 cells were treated with D-Galacturonic acid (D-GalA), D-Mannose (D-Man), L-Rhamnose (L-Rham), L-Arabinose (L-Ara), D-Galactose (D-Gal), D-Glucose (D-Glc), D-Xylose (D-Xyl) and sodium metaperiodate (NaIO_4_) for 1 h before being allowed to attach to 4 types of BC composites [BC, BCP (0.5 %), BCX (0.5 %), BCP (0.25 %) X (0.25 %)] for 20 min. Numbers of attached cells were enumerated as log CFU/cm^2^. Different uppercase letters indicate significant differences in bacterial attachment between types of composites (One-way ANOVA & Tukey’s pairwise comparison at *p* < 0.05). Different lowercase letters indicate significant differences in bacterial attachment within each type of composite whereas the absence of lowercase letters indicate no significant differences in bacterial attachment within each type of composite (One-way ANOVA & Tukey’s pairwise comparison at *p* < 0.05)

A higher concentration of D-galacturonic acid (2 % w/v) was used to further investigate its role in *Salmonella* attachment to the BC composites. The viability of all *Salmonella* strains were not affected (results not shown). The results showing the effect of monosaccharides and sodium metaperiodate on *Salmonella* attachment are summarized in Fig. 1. Compared to 1 % w/v D-galacturonic acid, 2 % w/v of the sugar significantly reduced the overall attachment of *S.* Enteritidis ATCC 13076 to all 4 types of BC composites (*p* < 0.05), whereas the overall attachment of *S. enterica* M4 to the 4 types of BC composites was significantly increased (*p* < 0.05). The overall attachment of *S.* Typhimurium ATCC 14028 to the BC composites was not significantly different at both sugar concentrations (*p* > 0.05) (Fig. 1). If D-galacturonic acid complements the attachment receptors on *Salmonella* cells, the sugar should specifically reduce the attachment of *Salmonella* cells only to BC composites which contain pectin. This was not the case as the attachment of *S.* Enteritidis ATCC 13076 and *S.* Typhimurium ATCC 14028 to the BC and BCX composites which did not contain pectin was also reduced significantly (*p* < 0.05) in some cases (Fig. 1). This suggests that D-galacturonic acid does not competitively block bacterial cell receptors and could have affected the bacterial cell in other ways (for example, non-specific adsorption to the bacterial surface), which has yet to be fully investigated. As the addition of sodium metaperiodate which cleaves the C-C bond between vicinal hydroxyl groups in sugars did not cause a significant decrease in the attachment of all strains (*p* > 0.05) (Fig. 1), this further confirmed suggestions that the attachment of *Salmonella* cells to BC composites is not likely to be mediated by specific binding of bacterial cell receptor to complementary sugar residues.

Some sugars significantly increased the attachment of the *S. enterica* strains (Fig. 1), for example, L-rhamnose and D-xylose significantly increased the attachment of *S.* Enteritidis ATCC 13076 and *S. enterica* M4 (*p* < 0.05). We propose that these sugar molecules could have increased the ability of the strains to auto-aggregate. Planktonic *Salmonella* cells in the bacterial suspension could have attached and aggregated onto the bacterial cells which were already attached to the composite surface. Sugars produced during gluconeogenesis have been shown to cause *S.* Typhimurium cells to aggregate [25]. Auto-aggregation assays could be carried out in future studies in order to test this under the conditions investigated in our study. In another study, Gram-negative bacterium *Azospirrilum brasilense* increased its capacity to aggregate in the presence of exopolysaccharide which consists of different sugars such as xylose, rhamnose and arabinose [26]. While these results are intriguing, we have yet to fully elucidate the mechanism of action of these sugars on *Salmonella* attachment in our study.

### Microscopy

We have shown that the attachment of *Salmonella* cells to the BC composites are unlikely to rely upon receptor-ligand interactions mediated by carbohydrates and bacterial surface adhesins. Their attachment to the BC composites were therefore most probably non-specific and stochastic in nature and instead could be governed by the physical properties of the attachment surface. Very few studies have investigated on the effect of varying levels of pectin and xyloglucan on the structural properties of BC-based PCW models. We have used three microscopic techniques to obtain structural information on the BC composites.

### Confocal laser scanning microscopy 

**Fig. 2:**
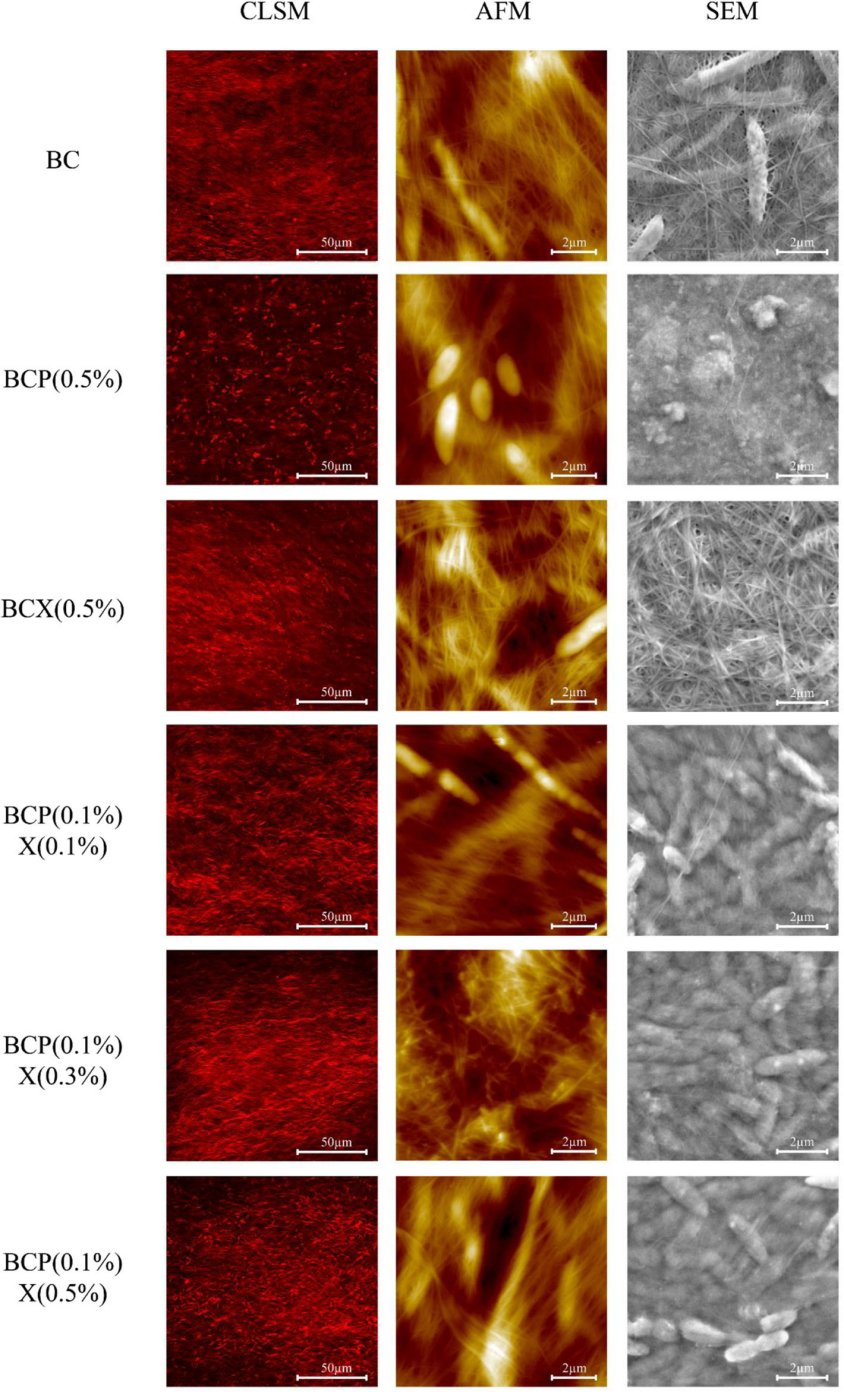
CLSM images, AFM height images and SEM images of the BC composites. Microscopy was carried out on BC, BCP (0.5 %), BCX (0.5 %), BCP (0.1 %) X (0.1 %), BCP (0.1 %) X (0.3 %) and BCP (0.1 %) X (0.5 %) composites which were harvested after 72 h growth in HS media with/without pectin and/or xyloglucan. For CLSM, cellulose fibrils and *Gluconacetobacter xylinus* cells appeared as *red* strands and bright *red* dots respectively after staining with Calcofluor White (CW). For AFM and SEM, strand-like cellulose fibrils and rod-shaped *Gluconacetobacter xylinus* cells were observed

CLSM images (Fig. 2) showed that the cellulose fibrils in all BC composites were tightly packed and appeared to be in a wavelike arrangement, creating gaps within the structure. Cellulose fibrils stained by CW appeared as red strands while *G. xylinus* cells which can also be stained by CW appear as bright red dots on the images. The BCP composite had a different appearance from the other BC composites with visible dark patches and very bright dots in its image. We suggest that the high amounts of pectin in the BCP composite coated the cellulose fibrils and fibrils were unable to take up the CW dye readily. Hence, bright red dots of *G. xylinus* cells which absorbed the CW dye and stained better than the fibrils were more obvious against the dark background.

**Fig. 3:**
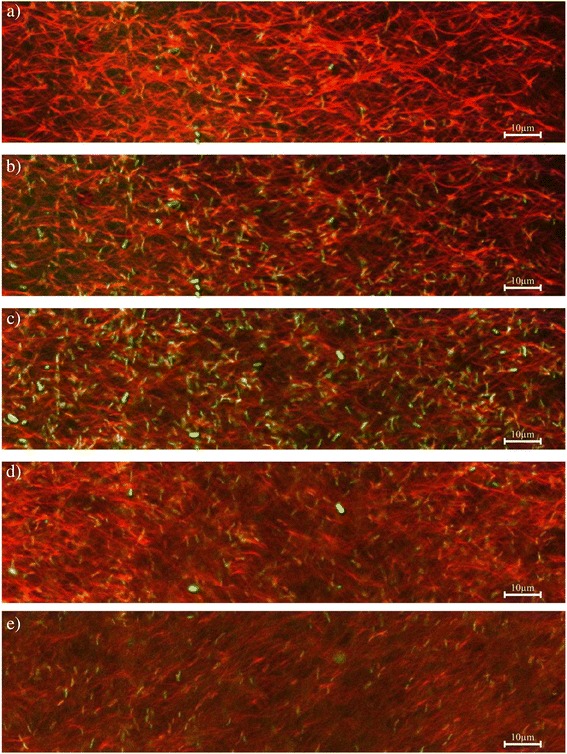
Internalization of *Salmonella* Typhimurium cells within the BCP (0.1 %) X (0.1 %) composite. Using the confocal laser scanning microscopy, *S.* Typhimurium cells dyed with Bromothymol Blue (shown in *green*) were observed to attach **a** on the BCPX surface (cellulose fibrils shown in *red*) and at **b** 2 μm, **c** 4 μm, **d** 6 μm and **e** 8 μm below the surface

In order to establish whether the *S.* Typhimurium ATCC 14028 cells could penetrate and attach inside the thick and tightly arranged BC composites, the *S.* Typhimurium cells were dyed with BTB and later allowed to attach to the BCPX composite. More *S.* Typhimurium ATCC 14028 cells attached inside the composite than at the surface, even up to a depth of 8 μm below composite surface (Fig. 3). This may be because *Salmonella* cells at the BC composites’ surface are exposed to shear force which can detach adhered bacteria, whereas bacteria attached within the composites were shielded from shear force [19].

### Atomic force microscopy (AFM)

Analysis of AFM height images (Fig. 2) showed that out of the 6 composites, BCP (0.1 %) X (0.3 %) had the thickest fibril diameter (180.0 ± 24.7 nm) and this was significantly higher than the other composites (*p* < 0.05) whereas BC had the thinnest fibrils (103.3 ± 10.4 nm) (*p* < 0.05) (Table 1). The imaged fibrils are macrofibrils consisting of bundles of microfibrils (<5 nm) strongly linked to one another. Our past results showed that *Salmonella* strains exhibited the highest attachment to the BCPX composite with 0.3 % (w/v) xyloglucan regardless of the amount of pectin added into the growth media [13]. The significantly thicker fibrils in the BCPX (0.3 %) composite may have provided a greater surface area for bacterial attachment.

**Table 1:** Measurements obtained from AFM height images of the BC composites

	BC	BCP (0.5 %)	BCX (0.5 %)	BCP (0.1 %) X (0.1 %)	BCP (0.1 %) X (0.3 %)	BCP (0.1 %) X (0.5 %)
Microfibril diameters (nm)	103.3 ± 10.4^a^	130.9 ± 17.2^b^	141.5 ± 16.6^b^	143.1 ± 23.6^b^	180.0 ± 24.7^c^	145.7 ± 25.6^b^
Average roughness (nm)	63.8 ± 23.2	79.1 ± 20.3	65.9 ± 12.3	77.2 ± 22.1	91.4 ± 24.3	73.9 ± 6.7
Root mean square roughness (nm)	75.1 ± 22.0	97.3 ± 24.8	76.5 ± 13.5	88.5 ± 23.9	110.6 ± 28.9	87.7 ± 9.7

Data are presented as mean ± SD where *n* = 30. Different lowercase letters indicate significant differences between types of BC composites whereas absence of lowercase letters indicate no significant differences between types of BC composites within the same row (One-way ANOVA & Tukey’s pairwise comparison at *p* < 0.05)

Although the addition of pectin and xyloglucan both increased fibril thickness, these polymers differed in the way they interact with cellulose fibrils. Formation of the BC-based PCW model has been shown to mimic the natural phenomenon of PCW deposition in native plants [27]. After synthesis in the Golgi apparatus, PCW polysaccharides such as pectin and xyloglucan are secreted separately into the extracellular matrix in plant cells [27]. In the native PCW, xyloglucan coats the cellulose microfibrils [7] while pectin forms a network around the cellulose-xyloglucan network [28]. In reference to this, we postulate that xyloglucan increases fibril diameter by forming strong cross-links with cellulose and coating the cellulose fibrils. Pectin increases fibril diameters as it also coats the fibrils but its primary function is to fill up the gaps between cellulose fibrils.

Showing similar results to our study, Fanta et al. [29] also measured cellulose fibril diameters of ~100 nm. They found that the fibril diameters of BC (110 ± 33 nm) and BCPX (123 ± 29 nm) were not significantly different from one another; but in contrast to our results, the fibril diameter for their BCP composite was much lower (45 ± 9 nm). These authors suggested that pectin may have resisted microfibril association but this was not apparent in our study. Another study by Cybulska et al. [24] supported our finding that cellulose fibrils for BCPX (~75 nm) were significantly thicker than both BC (~37 nm) and BCP (~46 nm). However, the difference in the size of cellulose fibrils between this study and ours may be related to the strains of *G. xylinus* used.

Another factor that could affect bacterial attachment is the surface roughness of the BC composites and this was measured from the AFM height images. Rough surfaces have greater surface area and more surface irregularities that provide favourable sites for bacterial adhesion [19]. We found that the average and RMS roughness were not significantly different between the BC composites, this suggests that surface roughness has no significant effect on the attachment of *Salmonella* cells to the composites. Similarly, Cybulska et al. [24] did not observe significant differences in RMS roughness between BC, BCP and BCPX composites.

**Fig. 4:**
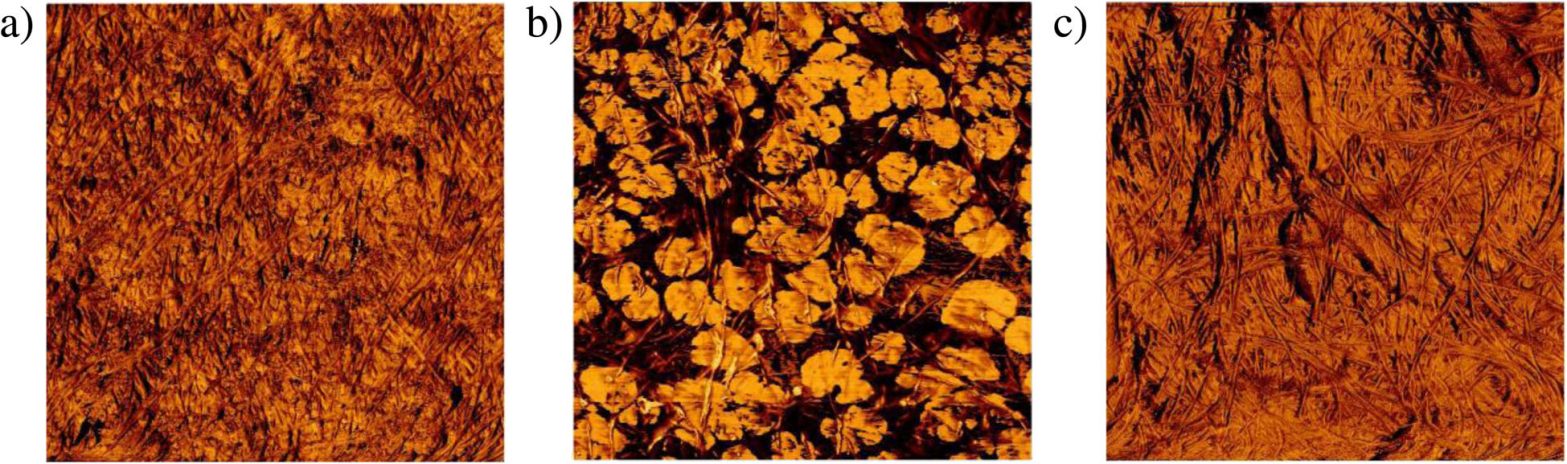
AFM phase images of the BCPX composites. Tapping mode was used to obtain AFM images of **a** BCP (0.1 %) X (0.1 %), **b** BCP (0.1 %) X (0.3 %) and **c** BCP (0.1 %) X (0.5 %)

AFM phase images give an indication of the sample hardness, chemical composition and elasticity [30]. Interestingly, the phase image of BCPX (0.3 %) exhibited more heterogeneity compared to the other BCPX (0.1 %) and BCPX (0.5 %) composites (as shown in Fig. 4) although chemical compositions of the 3 different BCPX composites were previously shown to be similar [13]. This suggests that pectin and xyloglucan interact differently at particular concentrations and cause distinct physical and structural changes to the composites which also influence bacterial attachment. It is still unclear how different concentrations of PCW components influence these specific structural changes, and whether surface mechanical properties have a role to play in bacterial cell adhesion, although this will be explored in future work.

### Scanning electron microscopy (SEM)

SEM was used to complement the results from AFM imaging. Unlike the AFM, SEM is able to analyse a large surface area and has a large depth of field which allows it to be used on relatively rough surfaces. Of the composites, the BC composite was clearly the most porous, showing the greatest textural variation, and the BCX composite was also more porous when compared to the others (Fig. 2). It was difficult to determine the porosity and pore size from the images, as the BC composites were too thick. Cybulska et al. [24] and Fanta et al. [29] both found that the BC has the highest porosity and BCP has lower porosity after the addition of pectin, whereas BCPX has the lowest porosity and greatest compactness amongst the composites.

According to Shah et al. [31], a porous BC matrix can easily trap liquid substances and small particles. Surface porosity has been found to favour bacterial attachment, probably due to the increased available area for attachment [32]. However, this was not the case in our previous study [13] as the more compact BCP and BCPX composites had a greater number of attached bacterial cells (more than 0.5 log CFU/cm^2^ higher) compared to the more porous BC and BCX composites. We hypothesize that pectin fills the voids between cellulose fibrils in the BCP and BCPX composites, and creates a complex matrix within the composites that can trap *Salmonella* cells that enter through the pores. Within the matrix, weak forces such as van der Waals forces, electrostatic forces and hydrophobic interactions [10] may mediate the initial reversible attachment. These weak forces draw bacterial cells closer to the attachment surfaces which then allow other stronger interactions to occur, such as covalent bonding, hydrogen bonding and cation bridging [11]. Although carbohydrates are generally considered to be highly polar, they still contain hydrophobic regions which allow non-specific hydrophobic bonding. Calcium bridges formed between free carboxyl groups of pectin chains [33] may favour the attachment of negatively charged *Salmonella* cells. On the other hand, fewer *Salmonella* cells (~2 μm) attach inside BC and BCX composites that do not contain the pectin matrix as they were able to swim more freely through their pores (~100 μm after 3 days incubation) [34].

Pectin masks cellulose fibrils and forms clumps on the surface of the BCP composite as can be seen in Fig. 2. Cellulose fibrils in the BC and BCX composites were more randomly arranged, while those in the BCPX composites appeared to be more unidirectional, however, individual fibrils cannot be distinguished clearly as they were coated with pectin and xyloglucan. Similarly, Cybulska et al. [24] also observed that microfibrils in the BCPX composite did not cluster in distinct bundles compared to those seen on the BC and BCP composites.

## Conclusions

This study showed that carbohydrate molecules did not selectively inhibit the attachment of *Salmonella* cells to BC-based PCW models. This suggests that the attachment of *Salmonella* cells to native PCWs were not mediated by receptor-ligand interactions involving carbohydrates and bacterial surface adhesins. Pectin and xyloglucan interact differently at varying concentrations which confer BC composites with distinct physical and structural characteristics that influence the extent of bacterial attachment to these surfaces. We posit that pectin fills in the voids between cellulose fibrils and reduces porosity but the pore sizes are sufficiently large to allow internalization of *Salmonella* cells while creating a matrix that is able to retain *Salmonella* cells. Xyloglucan increases cellulose fibril diameter and attachment surface, allowing even more *Salmonella* cells to attach when it is in association with pectin.

Overall, these results indicate that surface morphology is one of the key factors affecting bacterial adhesion to PCWs. The findings provide a key advancement in our understanding of how bacteria attach to PCWs, which will aid in the development of more effective fresh produce decontamination methods. This is important to prevent unnecessary produce waste, major economic losses to the produce industry and serious health consequences in many countries.

## Abbreviations

AC: Intermittent contact
AFM: Atomic force microscopy
ANOVA: Analysis of variance
ATCC: American type culture collection
BC: Bacterial cellulose
BCP: Bacterial cellulose-pectin
BCPX: Bacterial cellulose-pectin-xyloglucan
BCX: Bacterial cellulose-xyloglucan
BTB: Bromothymol blue
CLSM: Confocal laser scanning microscopy
CW: Calcofluor white
HS: Hestrin and Schramm
PBS: Phosphate buffered saline
PCW: Plant cell wall
RMS: Root mean square
SEM: Scanning electron microscopy
SPIP: Scanning probe image processor
SPSS: Statistical package for the social sciences
TSA: Tryptic soy agar
XLDA: Xylose lysine deoxycholate agar
